# Free-Energy Model of Emotion Potential: Modeling Arousal Potential as Information Content Induced by Complexity and Novelty

**DOI:** 10.3389/fncom.2021.698252

**Published:** 2021-11-19

**Authors:** Hideyoshi Yanagisawa

**Affiliations:** Design Engineering Laboratory, Department of Mechanical Engineering, The University of Tokyo, Tokyo, Japan

**Keywords:** emotion, free energy, bayes, gaussian generative models, arousal, uncertainty

## Abstract

Appropriate levels of arousal potential induce hedonic responses (i.e., emotional valence). However, the relationship between arousal potential and its factors (e.g., novelty, complexity, and uncertainty) have not been formalized. This paper proposes a mathematical model that explains emotional arousal using minimized free energy to represent information content processed in the brain after sensory stimuli are perceived and recognized (i.e., sensory surprisal). This work mathematically demonstrates that sensory surprisal represents the summation of information from novelty and uncertainty, and that the uncertainty converges to perceived complexity with sufficient sampling from a stimulus source. Novelty, uncertainty, and complexity all act as collative properties that form arousal potential. Analysis using a Gaussian generative model shows that the free energy is formed as a quadratic function of prediction errors based on the difference between prior expectation and peak of likelihood. The model predicts two interaction effects on free energy: that between prediction error and prior uncertainty (i.e., prior variance) and that between prediction error and sensory variance. A discussion on the potential of free energy as a mathematical principle is presented to explain emotion initiators. The model provides a general mathematical framework for understanding and predicting the emotions caused by novelty, uncertainty, and complexity. The mathematical model of arousal can help predict acceptable novelty and complexity based on a target population under different uncertainty levels mitigated by prior knowledge and experience.

## Introduction

**FIGURE 1:**
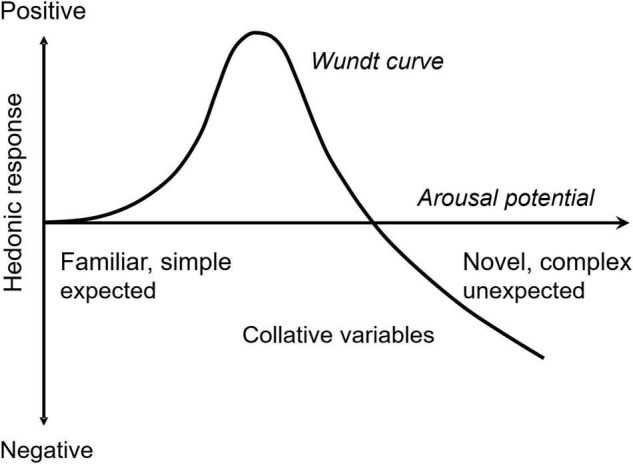
Hedonic function of arousal potential. Collative variables (e.g., novelty and complexity) are assumed to be the sources of arousal potential in Berlyne’s theory. An appropriate level of arousal potential maximizes positive hedonic response, whereas extreme arousal potential induces negative response.

Acceptance of novelty and complexity of incoming information depend on receivers’ emotions. Berlyne considered both novelty and complexity to be *collative properties* that are sources of arousal potential (Berlyne, 1970). He suggested that an appropriate level of arousal potential would induce a positive hedonic response, but extreme arousal potentials induce negative responses. The hedonic function of the arousal potential shapes an inverse U, the so-called *Wundt curve* (Figure 1). Arousal (i.e., intensity) and valence (i.e., positivity or negativity) are known to be dominant emotional dimensions (Russell, 1980; Lang, 1995). They comprise the *core affect* and are correlated with neural activity in specific brain regions (i.e., orbitofrontal cortex and amygdala, respectively) (Wilson-Mendenhall et al., 2013). The Wundt curve formulates a relationship between the core affect when the hedonic response is considered to be a valence component. In this sense, the Wundt curve is a valence function of arousal explaining the condition of positive acceptance of collative properties, such as novelty and complexity.

The mathematical formulation of arousal provides a precise prediction of the acceptance condition and the effect of certain factors of arousal in a general manner. Yanagisawa et al. formalized arousal using the Kullback–Leibler (KL) divergence (Kullback and Leibler, 1951) of the Bayesian posterior, termed *information gain*, and confirmed that it corresponds to human surprise *via* participants’ responses using event-related potential, P300, and subjective reports of surprise to novel stimuli (Yanagisawa et al., 2019; Ueda et al., 2021). The information gain is equivalent to Bayesian surprise representing visual salience (Itti and Baldi, 2009). Yanagisawa et al. formalized the Wudnt curve as a summation of reward and aversion functions, representing sigmoidal functions of the information gain (Yanagisawa et al., 2019; Miyamoto and Yanagisawa, 2021). Novelty, however, is only one of several sources of arousal potential (i.e., collative properties). Berlyne exemplified other collative variables, namely complexity and uncertainty. Experimental studies have shown that both complexity and novelty affect hedonic responses (valence) in art and music (Marin et al., 2016; Miyamoto and Yanagisawa, 2021), design aesthetics (Hekkert et al., 2003; Hung and Chen, 2012), and food preference (Giacalone et al., 2014). Information gain is content acquired from novelty; however, it does not involve complexity and uncertainty. To the best of my knowledge, a mathematical formulation of arousal potential caused by all these factors is yet to be established.

The present study proposes an arousal model that includes complexity and uncertainty as well as novelty. This model provides a general mathematical framework to explain arousal, the primary emotion dimension. Information content formulated as negative log probability of sensory stimuli has been hypothesized as being processed when one perceives sensory stimuli. In Section “Surprisal and Free Energy in Perception,” I mathematically demonstrate that information content is equivalent to minimized (variational) free energy or surprisal in perception. Free energy, which is originally a thermodynamic quantity in statistical physics, has recently been applied to the neuroscience field as a significant information quantity. Friston et al. proposed the free-energy principle that unifies brain theories (Friston, 2010). The principle of free-energy minimization mathematically explains cognitive components, such as perception and action (Friston et al., 2006). In this context, free energy represents a prediction error and uncertainties of signals in a Bayesian brain model (Knill and Pouget, 2004).

Recently, the free energy has been applied as a key information theoretic quantity to explain emotional states. Joffily and Coricelli (2013) formalized emotional valence as the rate of change of free-energy over time; they suggest that decreasing and increasing free energy corresponds to positive and negative emotion, respectively. The idea behind such a formulation is that emotional states reflect changes in the uncertainty about the somatic consequences of action (Wager et al., 2015; Seth and Friston, 2016; Clark et al., 2018). Hesp et al. (2021) formalized emotional valence as increased and decreased confidence (or precision) in one’s action model, termed *affective charge*. It is estimated as a higher metacognitive component of a deep hierarchical Bayesian model based on active inference that selects an action policy minimizing (expected) free energy. Although these studies formalize emotional valence and its dynamics, a formal representation of emotional arousal in relation to the free energy has not yet been proposed. Furthermore, the relationship between the free energy and emotional arousal characterized by collative properties (e.g., novelty and complexity) of sensory stimuli have not been elucidated.

In Section “Free Energy as Emotional Arousal Potential,” I mathematically demonstrate that free energy comprises the summation of information from both perceived novelty and perceived complexity of external stimuli and that this complexity is mathematically equivalent to minimized uncertainty. In Section “Analysis of Free Energy using a Gaussian Generative Model,” I derive a functional model of free energy using a Gaussian generative model. Using the function model, I predict how predictability (i.e., prior uncertainty and prediction error) and sensory-data variation (inverse precision) affect arousal potential. The model prediction of the predictability effect explains how personal knowledge and experience as factors of prior uncertainty change the optimal arousal potential (a peak of the Wundt curve, Figure 1). This helps us understand the personal differences caused by prior knowledge and experience, such as between novices and experts, on the preference of novelty and complexity. I demonstrate that the prior uncertainty effect is equivalent to the effect of sensory variance on arousal.

## Surprisal and Free Energy in Perception

The idea at the core of our model is that the total information content to be processed in the brain after perceiving sensory stimuli represents the potential cognitive load. This load functions as a source of emotional arousal (i.e., Berlyne’s arousal potential) that operates as an initiator of subsequent emotions. According to information theory (Shannon et al., 1949), information content is defined by the negative of the logarithm of probability (−log *p*), where *p* is an event’s probability. Therefore, I consider belief probability distributions in a situation where one obtains information content by perceiving the external world (e.g., physical properties like shape and color). Here, perception is defined as the estimation of the causes of sensory stimuli. Sensory stimuli are coded to neural activities in the brain (e.g., the firing rate of certain neuronal populations) *via* sensory organs (Yanagisawa, 2016). I term the neural codes as *sensory data* and assume the data to be a random variable, *x*, that follows a certain probability distribution. In our study, instead of true distributions, I assume that the brain has belief distributions, *p*(*x*), where *p*(*x*) is marginalized by the possible causes of sensory data represented as continuous random variables, θ ∈ *R* (e.g., physical properties (θ) like shape features and color that cause visual sensory data, *x*, *via* light).

I assume that a joint probability distribution between sensory data and its causes, *p*(*x*, θ), is learned based on past experiences of perceiving varied sensory data throughout one’s life. The statistical model is a generative model (Friston et al., 2006; Buckley et al., 2017). I assume that the generative model comprises observation model *p*(*x*|θ) and the prior *p*(θ).


(1) p(x,θ)=p(x|θ)p(θ).

With the generative model, one can predict sensory-data distributions under the assumption of a cause, θ. For example, one knows that the color “red” causes “this” sensory data from previous experience; therefore, one can imagine (generate) “red.” The exact Bayesian posterior, *p*(*x*|θ), represents perception of the cause, given data *x* (Knill and Pouget, 2004). This is usually difficult to compute. The variational Bayesian method then aims to find sufficient approximations of the exact posterior (Fox and Roberts, 2012). I assume an approximate posterior, *q*(θ), representing recognition density of a cause (Buckley et al., 2017). The deviation of *q*(θ) from the posterior *p*(θ|*x*) is evaluated using the KL divergence:


(2) $$D_{KL}\left(q\left(\theta \right)||p\left(x|\theta \right)\right)={\left\langle \ln q\left(\theta \right)-\ln p\left(x|\theta \right)\right\rangle }_{q\left(\theta \right)}.$$

I want to minimize the KL divergence to approximate the exact Bayesian posterior. However, the exact posterior is unknown. Here, I adopt the free-energy principle (Friston et al., 2006; Friston, 2010) to approximate the posterior by minimizing variational free energy in perception. The free-energy principle suggests that that any self-organizing system (i.e., brain) that is at equilibrium with its environment must minimize its free energy. It explains perception as the minimization of free energy, which comprises a joint occurrence of the sensory input and its cause (i.e., internal energy) and the negative entropy of the approximate distribution. It represents the difference between the recognition density and the generative model averaged over the recognition density in term of information (i.e., negative log probability).


(3) $$F=-{\left\langle \ln p\left(\theta ,x\right)\right\rangle }_{q\left(\theta \right)}+{\left\langle \ln q\left(\theta \right)\right\rangle }_{q\left(\theta \right)}.$$

With decomposition *p*(*x*, θ) = *p*(θ|*x*)*p*(*x*), the free energy is expressed as a summation of KL divergence and surprisal (Friston et al., 2006). The surprisal is the negative log of the marginalized likelihood representing model evidence, *p*(*x*) = ∫*p*(θ, *x*)*d*θ.


(4) $$F=D_{KL}\left(q\left(\theta \right)||p\left(x|\theta \right)\right)-\ln p\left(x\right).$$

Because surprisal is independent from the recognition density, and the KL divergence is greater than zero, the lower bound of free energy is surprisal: *F* ≥ −ln *p*(*x*). When recognition density is a sufficient approximation of the exact Bayesian posterior, the KL divergence becomes zero, and the free energy is equivalent to the surprisal. This approximation process is connected with perception using the recognition density according to the free-energy principle (Friston et al., 2006). Therefore, the surprisal corresponds to the free energy minimized by perceiving a sensory stimulus. Free energy is considered to represent information content that the brain potentially processes using recognition density to perceive sensory stimuli.

## Free Energy as Emotional Arousal Potential

### Novelty and Uncertainty

With another decomposition *p*(*x*, θ) = *p*(*x*|θ)*p*(θ), free energy is expressed as a summation of another two terms:


(5) $$F=D_{KL}\left(q\left(\theta \right)||p\left(\theta \right)\right)+{\left\langle -\ln p\left(x|\theta \right)\right\rangle }_{q\left(\theta \right)}.$$

When the recognition distribution is a sufficient approximation of the exact Bayesian posterior, *q*(θ) ≃ *p*(θ|*x*), the first term is equivalent to *Bayesian surprise*, and the KL diverges from posterior to prior. Bayesian surprise refers to the difference of prior information content from posterior information content averaged over posterior information (Itti and Baldi, 2009). It represents information content gained by perceiving sensory data *x*. In our previous study (Yanagisawa et al., 2019), we defined Bayesian surprise as information gain because it represents information content gained from incoming sensory data. I considered information gain to be an index of novelty and experimentally verified that it corresponds to human surprise (i.e., high arousal emotion) induced by unexpected and novel stimuli (Sekoguchi et al., 2019; Yanagisawa et al., 2019; Miyamoto and Yanagisawa, 2021; Ueda et al., 2021). Information gain refers to information content elicited by incongruity between prior expectation and posterior perception (Friston et al., 2015). Prior expectations are formed based on past experiences. One expects an event if it has been frequently observed in one’s life. Therefore, an unfamiliar event, which has rarely been observed in the past, causes incongruity between prior expectation and posterior perception and surprises the person.

The second term is the *inverse accuracy* (Penny et al., 2010; Friston et al., 2017). From the context of human cognition, the accuracy is how accurately one’s generative model prediction fits incoming sensory data, *x*. Thus, the term represents the extent to which the sensory data support the hypothetical estimates of the generative model in one’s brain. Lower accuracy implies greater uncertainty in the estimate. Someone who believes their own generative model perceives uncertainty of the cause of incoming sensory data when accuracy is low. Therefore, from a first-person perspective, the inverse accuracy is interpreted as the perceived *uncertainty* of the cause of the sensory data.

### Prior/Posterior Uncertainties and Unfamiliarity

The second term of free energy in Eq. (5), the inverse accuracy ⟨−ln *p*(*x*|θ)⟩_*q*(θ)_, represents the uncertainty of a cause. However, this uncertainty is distinguished from other types of uncertainties represented by variances (or inverse precisions) of signals. In Bayesian brain hypothesis (Knill and Pouget, 2004) and free energy principle (Feldman and Friston, 2010), the term “uncertainty” means the variances of neural signals. From that meaning of uncertainty, I define other types (i.e., *prior* and *posterior uncertainties*), which are represented by prior and posterior entropies, respectively, as uncertainties generated by precision of expectation or estimation:


(6) $$Prioruncertainty:{<-\ln p\left(\theta \right)>}_{p\left(\theta \right)},$$

(7) $$Posterioruncertainty:<-\ln p\left(\theta |x\right){>}_{p\left(\theta |x\right)}.$$

Prior uncertainty refers to the extent to which one’s belief of cause is uncertain before observing sensory data. For instance, if one is not familiar with paintings, one’s prior uncertainty about paintings is high. Conversely, if one learns about paintings and becomes familiar with them, one’s prior uncertainty decreases. During this learning process, the prior updates to the posterior, and the decrease of uncertainty averaged over the posterior is equivalent to the information gain (Bayesian surprise):


(8) $$D_{KL}\left(p\left(\theta |x\right)||p\left(\theta \right)\right)={\left\langle -\ln p\left(\theta \right)-\left(-\ln p\left(\theta |x\right)\right)\right\rangle }_{p\left(\theta |x\right)}.$$

Imagine that one is in an art gallery, but one does not know which exhibition one is in. One could form a generative model where θ corresponds to alternative categories of art, and *x* are the alternative paintings one could encounter. Upon observing a painting, *x*, one can update one’s prior beliefs about which exhibition one is in to one’s posterior beliefs about the exhibition (θ) one has found. In this situation, the more unfamiliar painting one observes, the more one is surprised and learned. Here, there are two kinds of unfamiliarity*: unfamiliarity of a category* (e.g., paintings as a category of art) and *unfamiliarity of an individual* (e.g., a specific painting). The former corresponds to prior/posterior uncertainty and the latter to information gain elicited by novelty or Bayesian surprise.

### Uncertainty Reduction in Bayesian Updating

The growing body of psychophysical evidence has shown that computations of perception, sensory motor control, and learning are Bayes’ optima that lead to the Bayesian coding hypothesis (Knill and Pouget, 2004; Körding and Wolpert, 2004). The free-energy principle explains the Bayesian coding hypothesis *via* free-energy minimization (Friston, 2010). Furthermore, a variety of animals in different ecological contexts behave in manners consistent with predictions of Bayesian updating models (Valone, 2006). Here, I demonstrate how an uncertainty (the second term of Eq. 5) minimizes in a Bayesian updating process.

Using the definition of conditional probability, *p*(*x*|θ)*p*(θ) = *p*(*x*)*p*(θ|*x*), the free-energy representation takes two forms: summation of Bayesian surprise and the uncertainty and a summation of KL divergence and surprisal.


(9) $$\begin{array}{c} F\left(x\right)=D_{KL}\left(q\left(\theta \right)||p\left(\theta \right)\right)+{\left\langle -\ln p\left(x|\theta \right)\right\rangle }_{q\left(\theta \right)}\\ \;=D_{KL}\left(q\left(\theta \right)||p\left(\theta |x\right)\right)-\ln p\left(x\right). \end{array}$$

Here, the uncertainty regards an expected surprise (i.e., the information content) of sensory data *x* given a cause, θ, −ln *p*(*x*|θ), averaged over a recognition distribution, *q*(θ). From Eq. 9, the uncertainty is a summation of three terms: KL divergence, (negative) Bayesian surprise, and surprisal:


(10) $$\begin{array}{c} U\left(x\right):={\left\langle -\ln p\left(x|\theta \right)\right\rangle }_{q\left(\theta \right)}\\ =D_{KL}\left(q\left(\theta \right)||p\left(\theta |x\right)\right)-D_{KL}\left(q\left(\theta \right)||p\left(\theta \right)\right)-\ln p\left(x\right). \end{array}$$

I assume that a recognition is set to a prior, *q*(θ) = *p*(θ), before obtaining data *x*. The uncertainty is equivalent to a summation of KL divergence and surprisal because the second term (i.e., Bayesian surprise) is zero.


(11) $$\begin{array}{c} U_{pri}:={\left\langle -\ln p\left(x|\theta \right)\right\rangle }_{p\left(\theta \right)}\\ =D_{KL}\left(p\left(\theta \right)||p\left(\theta |x\right)\right)-\ln p\left(x\right). \end{array}$$

In Bayesian estimation or recognition of θ given *x*, the recognition distribution is approximated to the posterior, *q*(θ) ≃ *p*(θ|*x*). As a result, the uncertainty is approximated to the summation of negative Bayesian surprise and surprisal because the first term, KL divergence, is approximated to be zero.


(12) $$\begin{array}{c} U_{post}:={\left\langle -\ln p\left(x|\theta \right)\right\rangle }_{p\left(\theta |x\right)}\\ =-D_{KL}\left(p\left(\theta |x\right)||p\left(\theta \right)\right)-\ln p\left(x\right). \end{array}$$

The difference in uncertainty before and after the recognition forms a summation of the KL divergence and the Bayesian surprise. The difference is always greater than zero because both KL divergence and Bayesian surprise are greater than zero.


(13) $$U_{pri}-U_{post}=D_{KL}\left(p\left(\theta \right)||p\left(\theta |x\right)\right)+D_{KL}\left(p\left(\theta |x\right)||p\left(\theta \right)\right)\geq 0.$$

Therefore, the uncertainty always decreases in the recognition process from the prior to the posterior, *q*(θ):*p*(θ) → *p*(θ|*x*).

The surprisal, the second term of Eq. 12, is a negative log likelihood marginalized with a prior, −ln *p*(*x*) = −ln⟨*p*(*x*|θ)⟩_*p*(θ)_. When a prior is updated to a posterior, *p*(θ):*p*(θ) → *p*(θ|*x*), the posterior uncertainty is equivalent to the surprisal of the model prediction with posterior; it corresponds to a negative log likelihood of a predictive distribution.


(14) $$U^{*}:=-\ln{\left\langle p\left(x|\theta \right)\right\rangle }_{p\left(\theta |x\right)}=-\ln p^{*}\left(x\right),$$

where p*(⋅) = ⟨*p*(⋅|θ)⟩_*p*(θ|*x*)_ is a predictive distribution. By applying Jensen’s inequality, I find that the updated uncertainty, U*, corresponds to the lower term of the uncertainty, *U*. Thus, the uncertainty decreases in the prior updating process.


(15) $$\begin{array}{c} U_{post}={\left\langle -\ln p\left(x|\theta \right)\right\rangle }_{p\left(\theta |x\right)}\\ \geq -\ln{\left\langle p\left(x|\theta \right)\right\rangle }_{p\left(\theta |x\right)}=U^{*}. \end{array}$$

Jensen’s inequity holds for any priors. Therefore, for any priors, the uncertainty is always decreased by Bayesian prior updating.


(16) $$\forall p\left(\theta \right),{\left\langle -\ln p\left(x|\theta \right)\right\rangle }_{p\left(\theta \right)}\geq \ln{\left\langle -p\left(x|\theta \right)\right\rangle }_{p\left(\theta \right)}.$$

Hence, the uncertainty is always decreased by both the recognition (perception) and the prior updating (learning) process. By alternately repeating the two processes and given the data from the same stimuli source, the uncertainty minimized to the surprisal of true distribution of data.

### Uncertainty Convergence to Perceived Complexity

Consider the case where one obtains *n* data *D^n^* with respect to *m* values, *x*_*i*_ ∈*R* (*i* = 1, …, *m*). The empirical probability for each value is *f*(*x*_*i*_), (*i* = 1, 2, …, *m*), where *f* is the empirical distribution of obtained data *x*. From Eq. 14, the lower-bound uncertainty of a prior updating is the negative log likelihood of the predictive distribution with respect to the data, *D^n^*. I find that the lower-bound uncertainty corresponds to the (*n* times) cross entropy (Chen et al., 2012).


(17) $$\begin{array}{c} U^{*}\left(D^{n}\right)=-\ln p^{*}\left(D^{n}\right)\\ =-\ln\prod_{i=1}^{m}p^{*}{\left(x_{i}\right)}^{n\cdot f\left(x_{i}\right)}\\ =-n\sum_{i=1}^{m}f\left(x_{i}\right)\ln p^{*}\left(x_{i}\right)\\ =n{\left\langle -\ln p^{*}\left(x\right)\right\rangle }_{f\left(x\right)}, \end{array}$$

where p*(*x*) = ⟨*p*(*x*|θ)⟩_*p*(θ|*D^n^*)_ is a predictive distribution given data *D^n^* of a random variable, *x*, and p*(*x*_*i*_) is the predictive probability of value *x*_*i*_. The cross entropy is a summation of a KL divergence and entropy.


(18) $${\left\langle -\ln p^{*}\left(x\right)\right\rangle }_{f\left(x\right)}=D_{KL}\left(f\left(x\right)||p^{*}\left(x\right)\right)+H\left(f\left(x\right)\right).$$

The KL divergence represents the deviation of the model prediction from the data (empirical) distributions, which is always greater than zero. Thus, the lower bound of the cross entropy is the entropy of the sample distribution.


(19) $${\left\langle -\ln p^{*}\left(x\right)\right\rangle }_{f\left(x\right)}\geq H\left(f\left(x\right)\right).$$

As discussed, the lower-bound uncertainty implies that the uncertainty after a prior is replaced with the Bayesian posterior; thus, the uncertainty monotonically decreases with the Bayesian updating of repeated data input given from the same stimuli source (i.e., a cause target of perception). Therefore, the cross-entropy converges to the entropy of the distribution of the data. In other words, when the model prediction (predictive distribution) fits the data distribution by updating the prior, the KL divergence is minimized to zero, and the cross-entropy approaches the entropy of the data distribution. By the statistical law of great numbers, the empirical distribution converges to the true distribution of the stimuli source. Therefore, after sufficiently observing a stimuli source (a target of perception or a cause of sensory data), the uncertainty converges to the entropy.

The entropy reflects the disorder or complexity of a data distribution. Therefore, the uncertainty minimized by Bayesian updating, given sufficient input data, is interpreted as the *perceived complexity* of the data. Previous studies have shown that the curvature entropy of a shape correlates to the complexity perceived by human evaluators (Ujiie et al., 2012; Okano et al., 2020). Those experimental results support the interpretation that the uncertainty minimized by observing sufficient data (i.e., evaluating a shape) represents perceived complexity.

### Summary

Table 1 shows a summary of free-energy decomposition and its meanings in the context of emotion arousal potential. Free energy, representing sensory surprisal, is equivalent to the summation of information gain or Bayesian surprise (e.g., novelty and incongruity). The inverse accuracy and the uncertainty of causes are the negative log-likelihoods weighted over recognition. After observing sufficient sensory data from a stimuli source, the uncertainty represents perceived complexity. Novelty, incongruity, complexity, and the uncertainty are the collative properties (i.e., sources of arousal potential) that Berlyne exemplified. This concordance suggests that the free energy explains the arousal potential induced by collative properties.

**TABLE 1:** Summary of semantics of free energy in emotion arousal potential.

Mathematical terms	Information contents	Arousal potential
−ln *p*(*x*) ≥ *nH*(*x*)	Free energy, surprisal, negative log likelihood	Arousal potential of collative variables
*D*_*KL*_(*q*(θ)||*p*(θ) ≥ 0	Bayesian surprise, information gain, prior/posterior uncertainty	Novelty, incongruity, unfamiliarity
⟨−ln *p*(*x*|θ)⟩_*q*(θ)_ ≥ *nH*(*x*)	Inverse accuracy entropy	Uncertainty, Perceived complexity

*Arousal potential is formulated as free energy minimized in perception: surprisal. Free energy comprises the summation of information gained from novelty, incongruity, and unfamiliarity and inverse accuracy representing perceived uncertainty and perceived complexity.*

## Analysis of Free Energy Using a Gaussian Generative Model

### Gaussian Free-energy Function

In the free-energy principle literature, Gaussian posterior, the so-called Laplace approximation, is assumed (Friston et al., 2006, 2007; Buckley et al., 2017). The limitation of this approximation is that it can be inaccurate when the mode is not near the majority of the probability mass. However, the Gaussian form is useful because the parameters provide an exact form of prediction errors (distance between means) and precisions (inverse variance). Here, I assume Gaussian distributions for the prior and posterior to analyze how predictability and sensory variance (i.e. inverse precisions) affect free energy and its components: the information gain and the uncertainty. Gaussian distribution is a probability distribution formed by two parameters: mean and variance. The difference in the mean of prior and likelihood represents prediction errors. Variances of the prior and the likelihood represent the prior uncertainty and the sensory variance, respectively. Hence, a Gaussian distribution can be used to explicitly analyze how predictability (prediction errors and prior uncertainty) and sensory variance affect free energy by partial differentiations of Gaussian parameters. To be simple, I assume that a causal state of stimuli does not change over time.

I assume that one obtains *n* sensory data samples, *x^n^* = (*x*_1_, *x*_2_, …, *x*_*n*_), from a stimulus source that conforms to a Gaussian distribution, *N*(*μ*, σ^2^), and has a generative model, *p*(*x*, *μ*) = *p*(*x*|*μ*)*p*(*μ*). Here, the mean (*μ*) represents a perception target (i.e., the cause of sensory data). When the conditional probability, *p*(*x*|*μ*), conforms to a normal distribution, the likelihood function of a cause (*μ*) given the sensory data is:


(20) $$\begin{array}{c} p\left(x^{n}|\mu \right)=\prod_{i=1}^{n}p\left(x_{i}|\mu \right)\\ =\prod_{i=1}^{n}\frac{1}{\sqrt{2\pi s_{l}}}\exp\left[-\frac{{\left(x_{i}-\mu \right)}^{2}}{2s_{l}}\right]\\ ={\left(\frac{1}{\sqrt{2\pi s_{l}}}\right)}^{n}\exp\left[-\frac{1}{2s_{l}}\left\{n{\left(\mu -\bar{x}\right)}^{2}+nS\right\}\right], \end{array}$$

where *s_l_* is the variance of *p*(*x*|*μ*) (i.e., sensory variance), $\bar{x}$ is the sample mean, and *S* is the sample variance. I assume that the sensory variance estimates the stimulus’s source variance, *s*_*l*_ ≃ σ^2^.

The sensory data’s free energy, using the generative model, is represented as follows:


(21) $$F\left(x^{n}\right)=D_{KL}\left(q\left(\mu \right)||p\left(\mu \right)\right)+{\left\langle -\ln p\left(x^{n}|\mu \right)\right\rangle }_{q\left(\mu \right)},$$

where *p*(*μ*) is a prior, and *q*(*μ*) is the recognition distribution of *μ*. When the prior follows the Gaussian distribution, *N*(η, *s*_*p*_), the Bayesian theorem estimates the posterior, *p*(*μ*|*x^n^*), that follows a Gaussian distribution, *N*(η_*post*_, *s*_*post*_), where


(22) $$Average:\eta_{post}=\frac{ns_{p}\bar{x}+s_{l}\eta }{ns_{p}+s_{l}},Variance:s_{post}=\frac{s_{p}s_{l}}{ns_{p}+s_{l}}.$$

When the recognition distribution is approximated to the Bayesian posterior, the free energy is expressed as a summation of the information gain (i.e., Bayesian surprise) and the uncertainty.


(23) $$\begin{array}{c} D_{KL}\left(N\left(\eta_{post},s_{post}\right)||N\left(\eta ,s_{p}\right)\right)+{\left\langle -\text{ln}p\left(x|\mu \right)\right\rangle }_{N\left(\eta_{post},s_{post}\right)}\\ =G+U. \end{array}$$

The information gain representing human surprise to novelty is derived as a quadratic function of prediction error, $\delta =\bar{x}-\eta$, which represents the difference between the prior mean and the likelihood peak, with coefficients as functions of prior variance (i.e., prior uncertainty), *s_p_*, and sensory variance, *s_l_* (Yanagisawa et al., 2019).


(24) $$\begin{array}{c} G_{n}=D_{KL}\left(N\left(\eta_{post},s_{post}\right)||N\left(\eta ,s_{p}\right)\right)=A_{G}\delta^{2}+B_{G}\\ A_{G}:=\frac{n^{2}s_{p}}{2{\left(ns_{p}+s_{l}\right)}^{2}},B_{G}:=-\frac{1}{2}\left(\text{ln}\frac{s_{l}}{ns_{p}+s_{l}}+\frac{ns_{p}}{ns_{p}+s_{l}}\right). \end{array}$$

The uncertainty is also derived as a quadratic function of prediction error (see the Appendix for the derivation).


(25) Un:=⟨-ln⁡p(x|μ)⟩N(ηpost,spost)=AUδ2+BUAU:=nsl2(nsp+sl)2,BU:=n2(spnsp+sl+ln 2πsl+Ssl).

Therefore, free energy, as the summation of the information gain (novelty) and the uncertainty of *μ*, is also a quadratic function of the prediction error with coefficients that are functions of the variances, *s_p_* and *s_l_*.


(26) $$\begin{array}{c} F_{n}:=G_{n}+U_{n}=A_{F}\delta^{2}+B_{F}\\ A_{F}:=\frac{1}{2}\frac{n}{ns_{p}+s_{l}},\\ B_{F}:=\frac{1}{2}\left\{\ln\left(ns_{p}+s_{l}\right)+\left(n-1\right)\ln s_{l}+n\ln2\pi +n\frac{S}{s_{l}}\right\}. \end{array}$$

### Convergence to Entropy and Perceived Complexity

Here, I demonstrate how the uncertainty converges to entropy to represent perceived complexity. A prior updated by Bayesian theorem with *n* samples follows *N*(η_*n*_, *s*_*n*_) with an average of $\eta_{n}=\left(ns_{p}\bar{x}+s_{l}\eta \right)/\left(ns_{p}+s_{l}\right)$ and a variance of *s*_*n*_ = *s*_*p*_*s*_*l*_/(*ns*_*p*_ + *s*_*l*_). Given additional *m* samples from the stimulus source, the posterior follows *N*(η_*n* + *m*_, *s*_*n* + *m*_) with an average of $\eta_{n+m}=\frac{\left(n+m\right)s_{p}\bar{x}+s_{l}\eta }{\left(n+m\right)s_{p}+s_{l}}$ and a variance of $s_{n+k}=\frac{s_{p}s_{l}}{\left(n+m\right)s_{p}+s_{l}}$. With the prior and posterior, the information gain (*G*_*n+m*_) and the uncertainty (*U*_*n+m*_) can be derived:


(27) $$\begin{array}{c} G_{n+m}:=D_{KL}\left(N\left(\eta_{n+m},s_{n+m}\right)||N\left(\eta_{n},s_{n}\right)\right)\\ =\frac{1}{2}[m^{2}\frac{s_{p}s_{l}}{ns_{p}+s_{l}}{\left(\frac{\delta^{2}}{\left(n+m\right)s_{p}+s_{l}}\right)}^{2}-\ln\frac{ns_{p}+s_{l}}{\left(n+m\right)s_{p}+s_{l}}+\\ \frac{ns_{p}+s_{l}}{\left(n+m\right)s_{p}+s_{l}}-1], \end{array}$$

(28) $$\begin{array}{c} U_{n+m}:=\left\langle -\ln p\left(x^{n+m}|\mu \right)\right)\rangle {}_{N\left(\eta_{n+m},s_{n+m}\right)}\\ =\frac{m}{2}\left[\frac{s_{l}}{{\left(\left(n+m\right)s_{p}+s_{l}\right)}^{2}}\delta^{2}+\frac{s_{l}}{\left(n+m\right)s_{p}+s_{l}}+\ln2\pi s_{l}+\frac{S}{s_{l}}\right]. \end{array}$$

When the sample number (*n*) is large enough (i.e., *n* → ∞), the information gain converges to zero, and the uncertainty converges to *m* times the entropy of the stimulus source population.


(29) $$\underset{n\to \infty }{\text{lim}}G_{n+m}=0,$$

(30) $$\underset{n\to \infty }{\text{lim}}U_{n+m}=\frac{m}{2}\left(\ln2\pi s_{l}+1\right)=mH\left(x\right),$$

where the entropy of the sensory data is:


(31) $$\begin{array}{c} H\left(x\right)=\int_{-\infty }^{\infty }-p\left(x\right)\ln p\left(x\right)dx\\ =\int_{-\infty }^{\infty }-N\left(\mu ,\sigma^{2}\right)\ln N\left(\mu ,\sigma^{2}\right)dx\\ =\frac{1}{2}\left(\ln2\pi \sigma^{2}+\frac{S}{\sigma^{2}}\right)≅\frac{1}{2}\left(\ln2\pi s_{l}+1\right)\\ ∵\sigma^{2}≅s_{l},\underset{n\to \infty }{\text{lim}}\frac{S}{\sigma^{2}}=1. \end{array}$$

Entropy is proportional to the logarithm of sensory variance, *H*(*x*)∝ln *s*_*l*_, when a Gaussian distribution is assumed. As discussed, the entropy of the sensory-data distributions represents the perceived complexity of the stimulus.

The free energy, as the summation of the information gain and the uncertainty, minimizes to entropy. In other words, $\underset{n\to \infty }{\text{lim}}F_{n+m}=\underset{n\to \infty }{\text{lim}}\left(G_{n+m}+U_{n+m}\right)=mH\left(x\right)$. By obtaining the sensory data, the information gain decreases to zero because the prior comes close to the true distribution of the stimuli source *via* Bayesian updates. The uncertainty decreases to the lower limit (i.e., entropy of the true distribution) because the predictive distribution comes close to the sample distributions, and the KL divergence becomes zero. With Gaussian posteriors and likelihoods, the uncertainty monotonically decreases as *n* increases. Therefore, when both information gain and the KL divergence between the predictive and data distribution converges to zero, free energy is minimized to the entropy of the data distribution.

### Effect of Predictability

Because the uncertainty is a quadratic function of prediction error, as shown in Eq. 25, deviations from prior expectations increase the uncertainty. Its gradient *A*_*U*_ is always positive and means the sensitivity of the effect of prediction error on the uncertainty. The gradient of *A*_*U*_ with respect to the prior variation *s*_*p*_ is always negative:


(32) $$\frac{\partial A_{U}}{\partial s_{p}}=\frac{-n^{2}s_{l}}{2{\left(ns_{p}+s_{l}\right)}^{3}}<0.$$

*s_p_* is proportional to the logarithm of prior entropy (e.g., prior uncertainty; see Eq. 6). Thus, a high prior uncertainty indicates that the prediction error’s effect on the uncertainty is low. In other words, a certain expectation (low prior variance) refers to a significant effect of prediction error on the uncertainty. By increasing the amount of data sampled, the gradient converges to zero, $\underset{n\to \infty }{\text{lim}}A_{U}=0$.

The gradient of *B*_*U*_ (the intercept) with respect to the prior variation *s*_*p*_ is always positive or zero:


(33) $$\frac{\partial B_{U}}{\partial s_{p}}=\frac{ns_{l}}{2{\left(ns_{p}+s_{l}\right)}^{2}}\geq 0.$$

Therefore, small or zero prediction errors and higher prior variations (i.e., prior uncertainty) lead to higher uncertainty. Conversely, large prediction errors and higher prior variation means smaller uncertainty, owing to greater gradients. By increasing the number of data samples, the intercept converges to the entropy times the sample number (i.e., infinite).


(34) $$\underset{n\to \infty }{\text{lim}}B_{U}=\frac{1}{2}\left(\ln2\pi s_{l}+1\right)\underset{n\to \infty }{\text{lim}}n=H\left(x\right)\underset{n\to \infty }{\text{lim}}n.$$

Hence, the intercept is dominated by the uncertainty when the sample is large.

Regarding the free energy function, Eq. 26, the gradient of *A*_*F*_ with respect to the prior variation (*s_p_*) is always negative:


(35) $$\frac{\partial A_{F}}{\partial s_{P}}=-\frac{n^{2}}{2{\left(ns_{p}+s_{l}\right)}^{2}}<0.$$

The gradient of *B*_*F*_ with respect to the prior variation (*s_p_*) is always positive:


(36) $$\frac{\partial B_{F}}{\partial s_{P}}=\frac{n}{2\left(ns_{p}+s_{l}\right)}>0.$$

**FIGURE 2:**
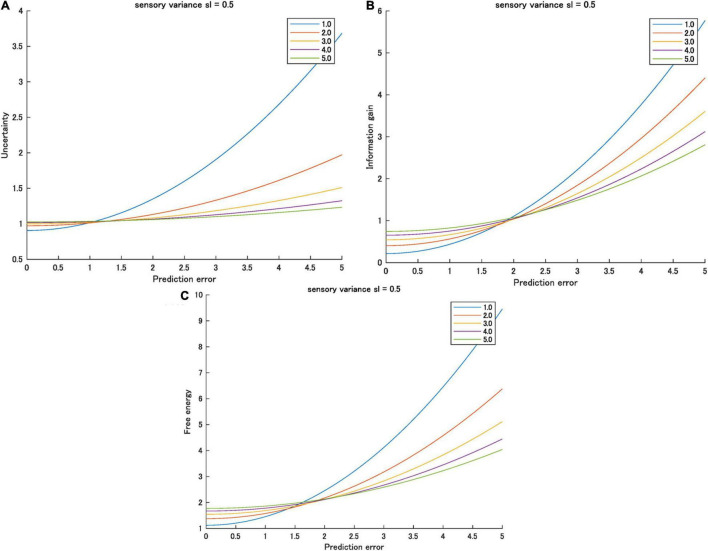
**(A)** Uncertainty, **(B)** information gain, and **(C)** free energy as functions of prediction errors for various prior variances ([1.0, 5.0]) when sensory valiance is 0.5. Interactions between prediction error and prior variance were observed for all information quantities.

Therefore, small or zero prediction errors and higher prior variations mean smaller free energy. Conversely, large prediction errors and higher prior variation means higher free energy. This inversion is observed in the effect of predictability on the information gain when *s*_*p*_ > *s*_*l*_ (Yanagisawa et al., 2019). Figure 2 shows an example of the inversion of information gain, uncertainty, and free energy as functions of prediction error varied by prior uncertainty.

### Effect of Sensory Variance

The entropy of sensory data is proportional to the logarithm of sensory variance (*s_l_*) as the estimate of sensory data population variation. With large data samples, *s_l_* comes close to the sensory data source variation. The entropies of sensory data sources represent perceived complexity. In this section, I analyze how the sensory variance affects the uncertainty, the information gain, and the free energy.

Regarding the uncertainty function, Eq. 25, the gradient of *B*_*U*_ with respect to sensory variance is always positive:


(37) ∂⁡BU∂⁡sl=n2sp2+sl2+slsp(nsp+sl)2≥0.

Therefore, zero prediction errors and higher data variances mean higher uncertainty. The gradient of *A*_*U*_ with respect to sensory variance depends on the magnitude relation of *ns_p_* and *s_l_*.


(38) $$\frac{\partial A_{U}}{\partial s_{l}}=\frac{n}{2}\frac{ns_{p}-s_{l}}{{\left(ns_{p}+s_{l}\right)}^{3}}.$$

**FIGURE 3:**
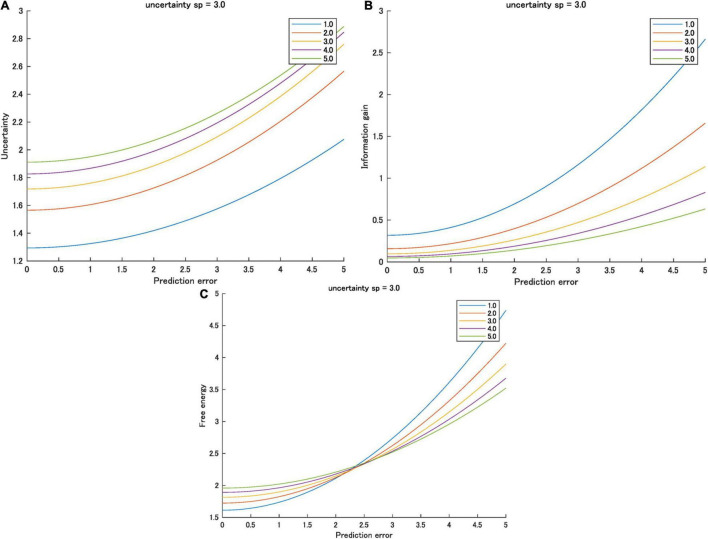
**(A)** Uncertainty, **(B)** information gain, and **(C)** free energy as functions of the prediction errors for various sensory variances ([1.0, 5.0]) when prior variance is 3.0. Interactions between prediction error and prior variance were observed only in free energy.

In most cases, the prior variance is greater than the likelihood variance, *ns*_*p*_ > *s*_*l*_, where *n* is a large natural number; therefore, partial derivative is greater than zero (i.e., the gradient of *A*_*U*_ is always positive). In this case, sensory variance increases *A*_*U*_. It also increases the uncertainty when the prediction error is greater than zero. Therefore, in most cases, *ns*_*p*_ > *s*_*l*_, and sensory variance and the uncertainty increase (Figure 3A).

Regarding the information gain function, Eq. 24, the gradient of both *A*_*G*_ and *B*_*G*_ with respect to the sensory variation is always negative:


(39) $$\frac{\partial A_{G}}{\partial s_{l}}=-\frac{n^{2}s_{p}\left(ns_{p}+s_{l}\right)}{{\left(ns_{p}+s_{l}\right)}^{4}}\leq 0,$$

(40) $$\frac{\partial B_{G}}{\partial s_{l}}=\frac{ns_{p}}{2}\left\{\frac{1-\left(ns_{p}+s_{l}\right)}{{\left(ns_{p}+s_{l}\right)}^{2}}\right\}.$$

Therefore, when (*ns*_*p*_ + *s*_*l*_) > 1, sensory variance decreases the information gain (Figure 3B). This is likely when *n* is a large natural number.

Regarding the free energy function, Eq. 26, the gradient of *A*_*F*_ with respect to the sensory variation (*s_l_*) is always negative:


(41) $$\frac{\partial A_{F}}{\partial s_{l}}=\frac{-n}{2\left(ns_{p}+s_{l}\right)}<0.$$

Therefore, sensory variance decreases the sensitivity of prediction errors on free energy. The gradient of *B*_*F*_ with respect to sensory variation (*s_l_*) is always positive:


(42) $$\frac{\partial B_{F}}{\partial s_{l}}=\frac{1}{2}\left(\frac{1}{ns_{p}+s_{l}}+\frac{n-1}{s_{l}}\right)>0.$$

Therefore, when the prediction error is zero, sensory variance increases the free energy. However, the gradient is higher when the sensory variance is smaller. As a result, when the prediction error is large, this relationship is reversed. The higher the sensory variance, the smaller the free energy (Figure 3C). Table 2 summarizes the effect of sensory variance on the effects of prediction errors on the three information quantities.

**TABLE 2:** Effect of sensory variance on *uncertainty*, *information gain*, and free energy and its condition.

Uncertainty		Information gain		Free energy	
Gradient	Intercept	Gradient	Intercept	Gradient	Intercept
increase	Increase (*ns*_*p*_ + *s*_*l*_) ≥ 1	decrease	Decrease (*s*_*p*_ > *s*_*l*_)	decrease	increase

*The gradient represents the sensitivity of prediction errors. The intercept is the effect when the prediction error is zero. Interaction of prediction error and sensory variance on free energy is predicted regardless any conditions.*

## Discussion

This study presents the relationship between the information content of perceived sensory stimuli (i.e., the free energy) and arousal potential (i.e., the potential of the primary emotional dimension). Information content to be processed after the perception of sensory stimuli (i.e., sensory surprisal) corresponds to the formulation of free energy commonly found in various disciplines, such as physics, statistics, and neuroscience (Friston et al., 2006). Free energy can be represented as a summation of the two terms of information content: information gain (Bayesian surprise) and surprisal (negative-log-likelihood averaged over the Bayesian posterior) (Eq. 10). Our previous study showed that the former represents novelty as a source of arousal potential (Yanagisawa et al., 2019; Ueda et al., 2021). I confirmed that the term also means expectation incongruity and unfamiliarity. The latter represents uncertainty of the estimated cause of sensory data. I demonstrated that the uncertainty decreases with the recognition and prior updating process based on Bayesian updating by adding sensory data from a stimuli source (i.e., a target of perception or cause of sensory data) in Section “Uncertainty Reduction in Bayesian Updating.” The uncertainty after the recognition and prior update (i.e., learning) is decomposed into KL divergence, representing deviation of sensory data from model prediction and entropy (Eq. 19). By reducing the model deviation to zero through iterations of Bayesian updating with enough data, the uncertainty minimizes to the entropy of sensory-data distribution as shown in Section “Uncertainty Convergence to Perceived Complexity.” From the analysis using the Gaussian generative model, I confirmed that the uncertainty converges to the entropy when sufficient data is provided in Section “Convergence to Entropy and Perceived Complexity.” Previous studies showed that the entropy represents the perceived complexity of a stimuli (Ujiie et al., 2012; Okano et al., 2020). Therefore, the uncertainty minimization by sufficiently observing a target of perception is considered to represent the perceived complexity.

All these factors (i.e., novelty, incongruity, unfamiliarity, uncertainty, and complexity) are collative variables exemplified by Berlyne as sources of arousal potential. Our previous model (Yanagisawa et al., 2019) considered only novelty. Hence, this model is more general than our previous model, including the second term regarding perceived complexity and uncertainty. Indeed, previous empirical studies have shown that the hedonic function of both perceived complexity and perceived novelty have inverse-U shapes. Mathematical formulations using free energy suggest that the sum of information content induced by novelty and complexity functions represents the arousal potential.

From the analysis of a Gaussian generative model, I found that all three information quantities (i.e., free energy, uncertainty, and information gain) are formulated as quadratic functions of prediction error, and the coefficients (i.e., gradient and intercept) are functions of prior variance representing prior uncertainty and sensory variance, which is the variance of the sensory-data distribution proportional to the entropy. Prediction errors increase all three information quantities. Prior variance decreases the effect that prediction errors have on these information quantities. In other words, the inverse prior variance functions as the sensitivity of prediction errors on the arousal potentials. However, the intercept increases as prior variance increases. Therefore, small or zero prediction errors mean lower prior variance, which in turn means lower information quantities.

This causes an interaction effect between the prior uncertainty and prediction error on the information contents. In our previous study (Yanagisawa et al., 2019), we experimentally verified the interaction effect on information gain, representing surprise to novelty using event-related potential, P300, and subjective reports of surprise from human participants. Free energy, representing arousal potential, (e.g., novelty, uncertainty, and complexity) involves the same interaction effect. Prior knowledge and experience decrease prior variance (i.e., prior uncertainty). Therefore, the more solidified the prior beliefs that one has from prior knowledge and experience, the less arousal potential that one elicits from events having small prediction errors. By contrast, the more certain prior belief one has, the greater arousal potential one elicits from an event with large prediction error. This characteristic explains the fact that prior knowledge and experience increase the acceptance of complexity (Tuorila et al., 1994; Silvia, 2005). Small prediction errors with certain prior uncertainties mean that one is familiar with the object (e.g., one is frequently exposed to the object), or one has sufficient knowledge about the object (i.e., one is an expert on the object). In such conditions, one’s information gain is small, and one has the capability to accept or enjoy more complex stimuli based on the Wundt curve shown in Figure 1.

I analyzed the effects of sensory variance on all three information quantities and summarized the results in Table 2. Sensory variance increases the uncertainty (intercept) and the sensitivity of prediction errors on the uncertainty (gradient). By contrast, sensory variance decreases information gain and the sensitivity of prediction errors on the information gain. Regarding the free energy (i.e., a summation of the uncertainty and the information gain), the sensory variance decreases the sensitivity of prediction errors (gradient), but it increases the intercept. When the prediction error is zero or small, sensory variance increases free energy (arousal potential). Conversely, when the prediction error is high, sensory variance decreases the free energy (arousal potential). This interaction effect is the same as the effect of the prior variance on the free energy (see Figures 2C, 3C). In conclusion, both the prior and sensory data are considered information sources or cues for estimating the cause of sensory stimuli in Bayesian perception (Knill and Pouget, 2004; Körding and Wolpert, 2004). Inverse variances represent the precision of each source. In this sense, it is reasonable that the effect of variance (inverse precision) of both sources on arousal potential is the same.

The model predictions shown in Figures 2, 3 provide a general scale of arousal (formalized as the free energy or surprisal) comprising novelty (the information gain) and perceived complexity (the uncertainty). This scale explains the difference in acceptable novelty and perceived complexity of stimuli based on predictability (i.e., prior uncertainty and prediction errors) caused by knowledge and experience (e.g., experts vs. novices) For a detailed analysis of the difference in acceptable novelty between experts and novices, see Miyamoto and Yanagisawa (2021). An experimental verification of this scale should be performed in future studies. This scale can help adjust to the preferable condition of novelty and complexity when designing artifacts (e.g., products and services) accepted by a target population (e.g., customers and users).

The human brain is an organ that processes significant amounts of information. This information involves a high cognitive load; therefore, a large amount of energy is required for processing. According to Friston’s free-energy minimization (Friston et al., 2006), the brain perceives the causes of sensory data such that the free energy is variationally minimized. Free-energy minimization equilibrium is a law that can be applied to both physical and biological systems. Surprisal corresponds to the minimized free energy and is concerned with the information content remaining after the perception of sensory stimuli (or recognition) is performed where the recognition distributions can be approximated to the Bayesian posterior. My expectation is that the minimized or remaining free energy can be used to activate emotional arousal and subsequent emotions, such as valence, and for free energy to be used as the general principle of emotion potential. This prediction supports the theory of a relationship between valence and free energy variation (Joffily and Coricelli, 2013).

Emotions motivate certain actions, such as approach and avoidance. Active inferencing suggests that the free energy can be reduced by acting to gain sensory evidence (Friston et al., 2015). This implies that emotions are functions that initiate actions for reducing the free energy, and the remaining free energy activates the said functions. In a model setting where an agent’s action changes hidden states, and the agent’s preference of sensory outcomes can be predefined as a prior, the confidence (or precision) of an agent’s action model or the notion of affective charge is adopted to estimate emotional valence as a state of metacognitive level of a deep hierarchical generative model (Hesp et al., 2021).

By contrast, the present study considered valence as a function of arousal potential characterized by collative properties (i.e., novelty and complexity) of sensory stimuli given a static cause (a hidden state), which does not change over time. See the analysis of Sections “Uncertainty Convergence to Perceived Complexity” and “Analysis of Free Energy Using a Gaussian Generative Model.” Preference to appropriate levels of arousal may be caused by curiosity and epistemic value in an active agent because these components comprise expected free energy (Friston et al., 2017), which is assumed to be arousal potential in the present study. A future direction to extend the arousal model should consider an active agent model that elucidates a mechanism of valence caused by arousal in a dynamic environment.

## Data Availability Statement

Publicly available datasets were analyzed in this study. This data can be found here: https://arxiv.org/abs/2003.10073.

## Author Contributions

HY designed and conducted the study and drafted and revised the manuscript.

## Conflict of Interest

The author declares that the research was conducted in the absence of any commercial or financial relationships that could be construed as a potential conflict of interest.

## Publisher’s Note

All claims expressed in this article are solely those of the authors and do not necessarily represent those of their affiliated organizations, or those of the publisher, the editors and the reviewers. Any product that may be evaluated in this article, or claim that may be made by its manufacturer, is not guaranteed or endorsed by the publisher.

## Appendix

Derivation of uncertainty using the Gaussian generative model:

$$\begin{array}{c} U={\left\langle -\ln p\left(x|\mu \right)\right\rangle }_{N\left(\eta_{post},s_{post}\right)}\\ =\int_{-\infty }^{\infty }-\ln\left[{\left(\frac{1}{\sqrt{2\pi s_{l}}}\right)}^{n}exp\left[-\frac{1}{2s_{l}}\left\{n{\left(\mu -\bar{x}\right)}^{2}+nS\right\}\right]\right]N\left(\eta_{post},s_{post}\right)d\mu \\ =\frac{n}{2}\left[\frac{1}{s_{l}}\int_{-\infty }^{\infty }n{\left(\mu -\bar{x}\right)}^{2}N\left(\eta_{post},s_{post}\right)d\mu +\ln2\pi s_{l}+\frac{S}{s_{l}}\right] \end{array}$$

With (μ-x¯)2=(μ-ηpost)2+2(ηpost-x¯)μ-ηpost2+x¯2, the integral is derived as

∫-∞∞(μ-x¯)2N(ηpost,spost)dμ=∫-∞∞(μ-ηpost)2N(ηpost,spost)dμ+2(ηpost-x¯)∫-∞∞μN(ηpost,spost)dμ+(x¯2-ηpost2)∫-∞∞N(ηpost,spost)dμ=spost+2(ηpost-x¯)ηpost+x¯2-ηpost2=spslnsp+sl+sl2(η-x¯)2(nsp+sl)2={spnsp+sl+sl(nsp+sl)2δ2}sl,

where prediction error $\delta =\eta -\bar{x}$. Therefore,

$$U=\frac{n}{2}\left[\frac{s_{l}}{{\left(ns_{p}+s_{l}\right)}^{2}}\delta^{2}+\frac{s_{p}}{ns_{p}+s_{l}}+\ln2\pi s_{l}+\frac{S}{s_{l}}\right].$$
